# A Case of Pulmonary Consumption, Cured by the Use of the Digitalis Purpurea: In a Letter from John Spence, M. D. of Dumfries (Virg.), to Dr. Mitchill, Dated Jan. 26, 1801

**Published:** 1808-06

**Authors:** 


*A Case of* PULMONARY CONSUMPTION, *cured by the Use of the* DIGITALIS PURPUREA: *In a Letter from* JOHN SPENCE, *M. D. of Dumfries (Virg.), to Dr.* MITCHILL, *dated Jan.* 26, 1801.

JAMES DRINAN, aged 31, a man of low stature, of a pale complexion, with a narrow chest and dark coloured hair, and by trade a shoemaker, June 18, 1800, labours under a severe cough, attended with a copious expectoration of gross, yellow coloured matter, which smells very offensively. The cough attacks him frequently during the day, but is most troublesome during the night, and in the mornings; feels little pain in his breast or side, except when he coughs; observes, when seized with a violent fit of coughing, that the matter he expectorates towards the close of the fit is tinged with blood, and tastes saltish; says he can distinctly trace the matter coming up from the short ribs on the right side; complains of being frequently very chilly, particularly in the evenings; and the chilliness he experiences has been so remarkable, that a few days ago, when Fahrenheit's thermometer stood nearly as high as 90 in the shade, he was obliged to cover himself with a blanket. These chills are succeeded by a high fever and profuse sweats. His appetite is bad, tongue foul, pulse hard and strong, and varies from 80 to 100 strokes, and upwards, in the minute; urine high coloured; is of a very costive habit

About the beginning of last month he was attacked with a pain in his right side, difficult breathing, and a very troublesome troublesome cough. For these complaints he was bled and blistered twice, took cooling purges, James's powders, and, in every respect, pursued attentively the antiphlogistic course. All his symptoms were thereby relieved, excepting his cough, which was harassing. About the 1st of June he suddenly expectorated a great deal of matter, the odour of which was excessively disagreeable, insomuch that scarcely any person could stay in the room with him. On the 10th of the same month all his symptoms became worse, accompanied with rigours, which were followed by fever and wasting sweats. All this time he adhered rigidly to a low diet, living almost entirely on strawberries and milk, and rode out every day. When he rode, he did not suffer his horse to go out of a walking pace. He likewise, by the advice of a neighbour, drank a great deal of horehound tea. Notwithstanding, however, all his care, debility and emaciation of body became daily more alarming; and so restless were his nights, and distressing his cough, that he was obliged to have recourse to opiates in large doses. From these circumstances, as well as from his profuse sweats and great expectoration, he was so convinced that his dissolution was rapidly approaching, that he deliberately settled his affairs; and he was the more disposed to give into this persuasion, as most of his relations had gone off, he said, in the same way.

He has resided several years in Dumfries (Virginia), and conducted himself with great sobriety and industry. He has generally enjoyed good health, except that he has frequently been attacked with slight catarrhal affections; and then his cough, though troublesome, was never dangerous. Although a shoemaker by trade, he has not, for many years, been engaged in the laborious part of that business, having a shop of his own, with a great number of journeymen, for whom his sole employment is to cut out work. —To-day I advised him to try the digitalis; and he very cheerfully embraced my proposal.

I directed him to begin with thirty drops of the saturated tincture in a wine glassful of mint tea, and to repeat this dose every forenoon and afternoon. At the same time I directed him to ride out every morning if able, and still to adhere to the antiphlogistic regimen. He was likewise directed to keep his bowels open by means of aloetic pills.

June 20. He took his medicine yesterday, as directed. No nausea excited by it, nor any change in his pulse. To-day he took forty drops of the tincture, and soon after rode out, when, unfortunately, his horse ran off, and threw him; him; in consequence of which he has been attacked with very sharp pains in his side, and a severe spitting of blood, for which he has been copiously bled.

21st. He has had a bad night; has expectorated a great deal of blood during the night; and this morning, he says, he coughed up a vast quantity of corruption, of a most offensive nature. As he feels no uneasiness from the drops he insists upon taking them in much larger doses, observing, that no time is to be lost. Six o'clock, P. M. Has taken eighty drops of the tincture twice to-day, without exciting nausea; his pulse, however, is now as low as 66 strokes in a minute.

22d. Sweated profusely last night; slept ill; has coughed up a good deal of matter this morning.—Seven o'clock P. M. Has taken 100 drops twice to-day. No nausea; pulse about 60; cough not so troublesome. Again rides out.

23d. Six o'clock, P. M. Having been obliged to visit a patient in the country to-day, he had the boldness to venture upon three doses of the tincture, 140 drops in each dose, in my absence. No nausea, however, was produced by the medicine; but he observed his pulse as low as 54 and 58. Says he has felt no pain in his side. His cough is easy, but what he expectorates is still offensive. Directed not to exceed 110 drops at a time.

24th. Seven o'clock, P. M. Has taken two doses of 110 drops to-day. No nausea; pulse as yesterday; sleeps rather better.

25th. Seven o'clock, P. M. Took 110 drops at half past eight o'clock this morning, and another dose at half past twelve; soon after which he felt very sick, and sent for me. I found him in bed, complaining of great nausea; and it was with difficulty he could prevent himself from puking. His skin was very cool, and his pulse beat precisely 45, and was full and soft; and at this hour (seven o'clock in the evening) it continues to beat nearly the same strokes in the minute.

27th. Took no drops yesterday, as the nausea continued; and his pulse did not exceed 50. No fever nor sweat last night. Observes that he makes a great deal of water; and, what he thinks extraordinary, from being of the colour of brine, it is now perfectly clear. His stools too, he observes, are so dry, that they crumble to pieces like dry clay. His diet still continues of milk and vegetables, and he indulges freely in ripe fruit. Has been obliged to take opening pills frequently.

28th. Slept well; has coughed very little this morning; took took only one dose of 100 drops of the tincture yesterday, which occasioned a distressing nausea all day, and kept his pulse low. There is an eruption all over his body, like the prickly heat.

29th. Has passed a good night; no return of any pain in his breast or side; little cough; little expectoration, and not offensive. Took three doses of the tincture yesterday, 100 drops in each dose, without immediately exciting nausea; but this morning he has been very sick at his stomach. Thinks this nausea relieved by drinking a little hyson tea; and had a keen appetite for his breakfast, which is remarkable. About half an hour before he got out of bed to-day he counted his pulse accurately, when it beat only 38 strokes in a minute; and at this hour (ten A. M ) though he has been walking about, it beats exactly 50, and is very soft. Omit the tincture to-day.

July 1. Has slept well these two nights. Continues to mend. Rode eight miles this morning. Takes 100 drops of the tincture once a day, which keeps his pulse under 60. He is directed to ride out every morning, to avoid all bodily exertions, to continue his vegetable diet, and to take the drops only every second or third day, or what is just sufficient to keep his pulse moderate.

20th. He has been taking the fox-glove as directed, but finds himself so entirely relieved from all his pectoral complaints that he begins to leave it off. From the 22d of June to this date his pulse has never exceeded 50, excepting at one time, when he imprudently engaged in very active exercise.

Nov. 1. Since last report he has enjoyed perfect health. For ten weeks past he has taken none of the digitalis, and now eats meat freely. Of late he has been remarkably drowsy, and says it requires some exertion to keep awake. Since he began the fox-glove he has taken no preparation of opium; indeed, no other medicine except aloetic pills occasionally.

January 24, 1801. He continues to enjoy good health, and, at present, is fatter than he has been for many years.

The tincture used in the above case was prepared as follows: Two ounces of fox-glove, nicely dried, were coarsely powdered, and infused for forty-eight hours in half a pint of French brandy, and then passed through filtering paper.

